# A Treatise on Amputations of the Extremities and Their Complication

**Published:** 1885-08

**Authors:** 


**A Treatise on Amputations of the Extremities and their Complication.** By B. A. WATSON, A.M., M.D., Surgeon to the Jersey City Charity Hospital, to St. Francis, and Christ Hospitals in Jersey City, N. J., &c. Illustrated by upwards of 240 engravings, and two full-page plates. Price, $5.50. Philadelphia: P. Blakiston, Son & Co. 1012 Walnut street. 1885.

The present work aims to discuss all the questions directly or indirectly pertaining to the subject of amputations and their complications. He dedicates the work to Lister, the father of antiseptic surgery, and, therefore, places himself on the side of this most important advance in surgery. Dr. Watson brings to his task a large experience in military and civil life, and is an enthusiastic student, embodying in his labor the most recent advances on the subject made by European and American surgeons. We think the work is a valuable contribution to surgery, and commend it to our readers whose tastes and opportunities lead them into this important department of their profession.

